# A Non-catalytic Deep Desulphurization Process using Hydrodynamic Cavitation

**DOI:** 10.1038/srep33021

**Published:** 2016-09-08

**Authors:** Nalinee B. Suryawanshi, Vinay M. Bhandari, Laxmi Gayatri Sorokhaibam, Vivek V. Ranade

**Affiliations:** 1 Academy of Scientific and Innovative Research (AcSIR), CSIR-National Chemical Laboratory, Pune, 411008, India; 2 CSIR-National Chemical Laboratory, Chemical Engineering & Process Development Division, Pune, 411008, India; 3 Visvesvaraya National Institute of Technology, Applied Chemistry Department, Nagpur, 440010, India

## Abstract

A novel approach is developed for desulphurization of fuels or organics without use of catalyst. In this process, organic and aqueous phases are mixed in a predefined manner under ambient conditions and passed through a cavitating device. Vapor cavities formed in the cavitating device are then collapsed which generate (*in-situ*) oxidizing species which react with the sulphur moiety resulting in the removal of sulphur from the organic phase. In this work, vortex diode was used as a cavitating device. Three organic solvents (n-octane, toluene and n-octanol) containing known amount of a model sulphur compound (thiophene) up to initial concentrations of 500 ppm were used to verify the proposed method. A very high removal of sulphur content to the extent of 100% was demonstrated. The nature of organic phase and the ratio of aqueous to organic phase were found to be the most important process parameters. The results were also verified and substantiated using commercial diesel as a solvent. The developed process has great potential for deep of various organics, in general, and for transportation fuels, in particular.

Deep desulphurization or reducing sulphur content of various organics, in general, and transportation fuels, in particular to very low level (~10 ppm) is a demanding but essential operation, especially from pollution point of view. In the case of transportation fuels, Governments worldwide mandate sulphur concentration to the level of 15 ppmw and 30 ppmw in diesel and gasoline respectively^1^,^2^,^3^. For fuel cell applications, the sulphur levels are required to be much lower (less than 1 ppmw) to avoid poisoning of the catalyst. For organics such as turpentine, removal of sulphur can be important in organic synthesis such as production of camphor.

Deep desulphurization during petroleum refining operations has been a challenge mainly due to the difficulties associated with removal of refractory sulphur compounds and also due to varying nature of compounds in different fuel fractions^4^. A catalytic process of hydrodesulphurization (HDS) commonly used in refineries, is considered as a satisfactory method for lowering sulphur content up to 350 ppm and thereafter pose limitations due to low reactivity of the remaining refractory compounds and increased cost of operation^5^,^6^. The conventional HDS process employs catalyst such as Co-Mo or Ni-Mo, requires high temperatures of the order of 450 °C, and high pressures of the order of 20–40 atm. Employing HDS process to meet the new standards is expected to require 3 fold increase in the catalyst volume/reactor size adversely affecting economic viability apart from adversely affecting quality of fuel^4^. A number of alternative processes have been investigated which include adsorptive desulphurization^1^,^7^,^8^,^9^,^10^,^11^, biodesulphurization^4^,^12^ and oxidative desulphurization^13^,^14^. Adsorptive deep desulphurization appears to hold promise and a number of sorbents starting from simple activated carbons^15^ to *π*-complexation adsorbents, where Cu-Y and Ag-Y zeolites were shown to exhibit good capacity for thiophene sulphur removal from benzene and n-octane mixtures^3^,^16^. Recently, a more complex process by combining oxidation and extraction has been reported– extractive and catalytic oxidative desulphurization (ECOD) that employs catalyst for oxidation and suitable extractant for removing oxidised products as an alternative to the existing HDS process^17^,^18^. Acid treated activated carbons have also been considered to promote oxidation in oxidative desulphurization^19^.

There are some reports of using cavitation coupled with catalysts for desulphurization. Ultrasound assisted oxidative desulphurization has been discussed to a certain extent essentially in presence of various catalysts^20^,^21^,^22^,^23^. Jin *et al*.^24^ reported sulphur removal in coal tar pitch by oxidation with hydrogen peroxide using trichloroacetic acid as catalyst and using ultrasound waves. A U.S. patent^25^ discloses process with hydrodynamic cavitation-catalyzed oxidation of sulphur-containing substances that requires presence of at least one oxidizing agent like hydrogen peroxide. Thiophene has been considered to be one of the most difficult and refractory organic sulphur compounds in oxidative desulphurization as compared to benzothiophene and other sulphur derivatives^26^,^27^.

In this work, we present a new process based on hydrodynamic cavitation for deep desulphurization of fuels and organics without employing any catalyst and under mild operating conditions. Thiophene was chosen as a model sulphur compound mainly due to limitation of conventional oxidation processes in its removal. We believe that the presented results demonstrate the applicability of proposed method based on hydrodynamic cavitation for sulphur removal and demonstrate its great potential for deep desulphurization of various organics, in general, and for transportation fuels, in particular.

## The Process: Desulphurization using Hydrodynamic Cavitation with Vortex Diode as Cavitating Device

**Figure 1:**
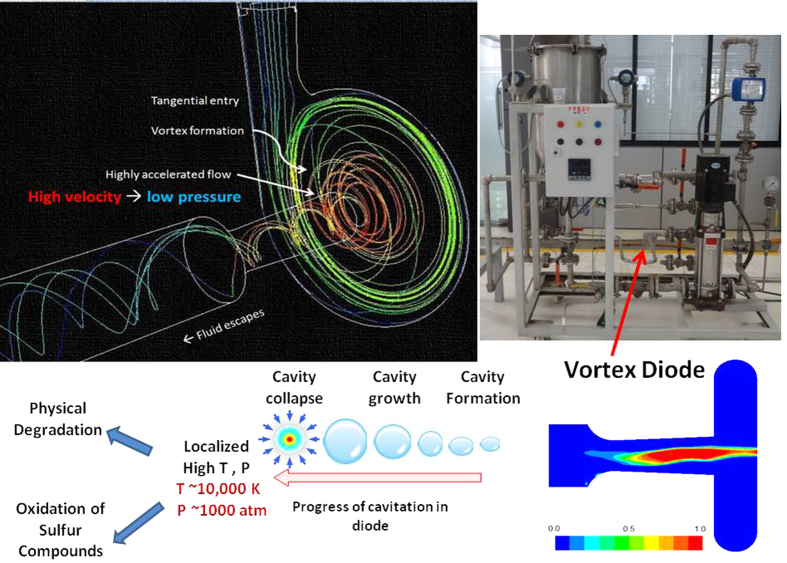
Schematic of Vortex Diode and Cavitation Process.

In the proposed approach, sulphur containing organic phase is mixed with water under ambient conditions and passed through a vortex diode^28^. Vapor cavities are formed in the diode and are transported to the downstream region where these cavities collapse. The cavity collapse generates localized very high pressure and temperature^29^ as well as hydroxyl radicals. Interaction of hydroxyl radicals under these locally extreme conditions result in removal of sulphur from the organic phase without any catalyst under apparently ambient conditions of bulk. A schematic of vortex diode functioning, possible steps in desulphurization using cavitation process and photograph of the experimental set-up for carrying out the experiments of the developed process are shown in Fig. 1. Details of experiments are discussed in the following sections. Key results and some comments on possible application for large scale sulphur removal are included after that.

## Experimental

### Materials and Methods

AR grade Thiophene (Sigma-Aldrich, >99%) was used as a model sulphur compound. n-Octane (Loba chemie, 98%), n-Octanol (Loba chemie, 99%), Toluene (Merck, >99%) were used as organic medium and for preparing model fuel. A commercial diesel was obtained locally. Sulphur analysis was carried out on Total Sulphur analyser TN-TS 3000 (Thermoelectron Corporation, Netherlands) and Gas chromatograph (Agilent 7890A) equipped with CPSil 5CB for sulphur as column (30 m × 320 *μ*m × 4 *μ*m) in conjunction with flame photometric detector (FPD). Helium was used as a carrier gas with flow rate of 2 mL/min and split ratio of 10:1 (20 mL/min flow rate). The injector temperature employed was 250 °C with injection volume of 0.2 *μ*L and total analysis time of 25 min. The oven temperature was ramped at 20 °C/min from 40 °C to 100 °C and at 60 °C/min from 100 °C to 230 °C. Reproducibility of the experimental results was checked and was found satisfactory.

### Experimental set-up

**Figure 2:**
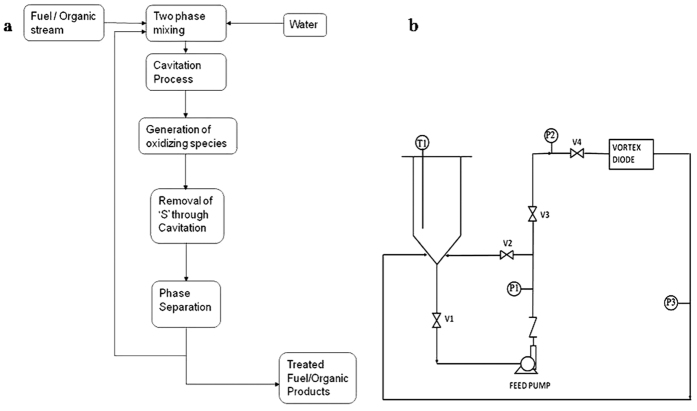
Block diagram of the process and Schematic of experimental set-up.

A schematic of experimental set-up for deep desulphurization using hydrodynamic cavitation is shown in Fig. 2. The experimental data was collected on this set-up using vortex diode as a cavitating device (nominal rated capacity, 1 m^3^/h). The setup has a holding tank of 60 L capacity, high pressure vertical multistage centrifugal pump (China Nanfang Pump, Model CDLF 2-17; SS 316, 1000 LPH at 152 MWC, 2.2 kW, 2900 rpm, 415 V AC, 3 phase, 50 Hz motor), control valves, and cavitation reactors. The flow through vortex diode was controlled using the bypass valve. Temperature was maintained using cooling coils inserted in the tank and with cooling system (JULABO, FP50). Flow transmitter (KROHNE, H250), pressure transmitters (Honeywell, ST700), Resistance Temperature Detector (RTD) (Eureka Engg. Enterprises, India) were used for the measurements. The entire set-up was fabricated in SS-316.

A known volume of 12 to 20 L was used for each experiment by appropriately measuring the organic and aqueous phase volumes. Initial sulphur concentration in the feed was adjusted to predetermined concentration. Experiments were carried out to evaluate effect of various parameters such as pressure drop, initial concentration, ratio of organic to aqueous phase (in terms of % organic phase) etc. Samples were collected at regular intervals of time and sulphur content was analysed in the organic phase using TN-TS 3000 Total sulphur analyser. The results were also cross-checked using gas chromatograph with FPD for sulphur analysis.

Some experiments were also carried out to ensure that sulphur removal is not via extraction. For establishing this, sulphur containing organic phase was mixed vigorously with liquid-water in agitated vessels (stirred tank with turbine impeller, rpm > 200) and samples were analysed at regular time intervals. The samples were also checked for changes in pH and COD using Spectralab MP-5 pH meter and Spectroquant Pharo 100 spectrophotometer (Merck Limited) where Spectroquant TR 320 was used as digester for digestion of samples for 2 h at 148 °C.

## Results and Discussion

### Hydrodynamic cavitation for sulphur removal

**Figure 3:**
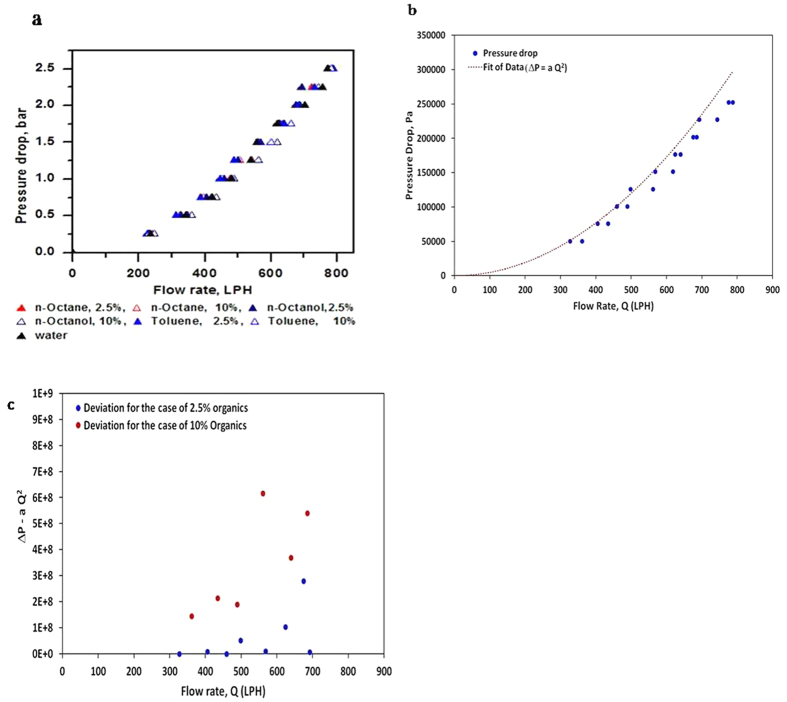
Inception of Cavitation; (**a**) Effect of solvent; (**b**) Calculations to demonstrate cavitation occurring at a ΔP of 0.5 bar; (**c**) Prediction of inception of cavitation based on deviation from square law.

Initial experiments were carried out to identify point of cavitation inception. Pressure drop measurements as a function of flow rate of two phase mixture (organic phase and water) were carried out. The contribution of bypass valve in the overall cavitation can be considered to be insignificant due to its partial closing in most cases (especially at very low pressure drop across vortex diode) and also due to the fact that mere cavity formation is not sufficient and requires effective cavity collapse for cavitation phenomenon as depicted in Fig. 1. It was established in earlier studies that cavitation inception can be identified from the deviation of measured pressure drop from the usual square law (ΔP proportional to square of flow rate or mean velocity). It was established that for the case of octanol – water mixture (up to 10% volume percent of octanol), the cavitation inception occurs just before the pressure drop across vortex diode reaches 0.5 bar. The details of pressure drop and identification of inception point are provided in Fig. 3. All the further experiments were carried out at two values of pressure drop across vortex diode (0.5 bar and 2 bar with flow rate of ~330 and 680 LPH respectively).

The initial sulphur content of organic phase (e.g. octanol) was adjusted to predetermined concentration (e.g. 300 ppm) by adding appropriate quantity of thiophene. The thiophene containing organic solvent was mixed with measured quantity of water to generate two phase mixtures with organic solvent volume fraction of 2.5% and 10%. The sulphur content in organic phase (obtained by separating organic layer from the treated mixture) was monitored as a function of time. The experiments were carried out to quantitatively understand influence of various process parameters. Some of these results are discussed in the following to elucidate influence of key process parameters.

### Influence of pressure drop, initial concentration and volume fraction

**Figure 4:**
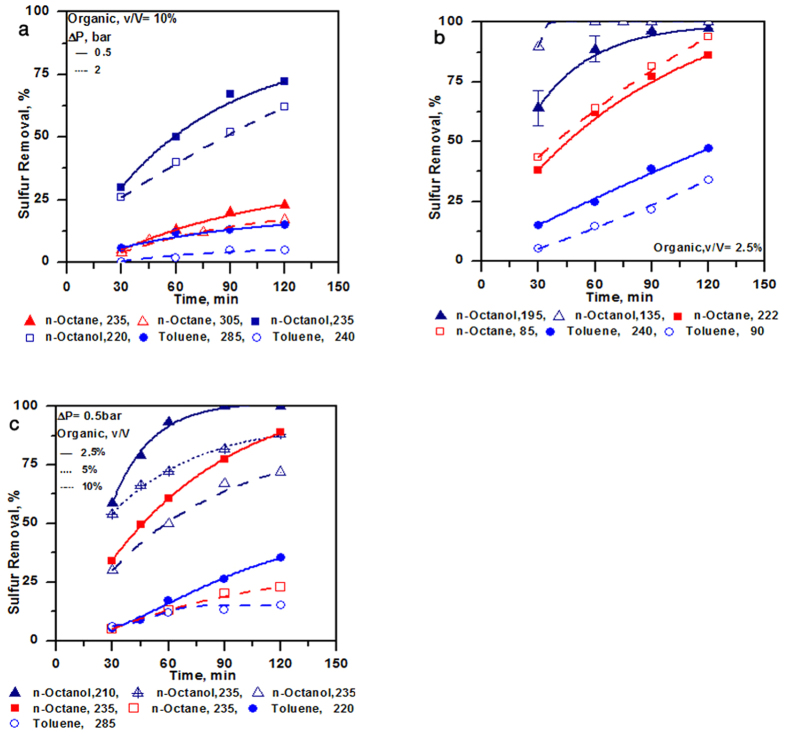
Effect of different parameters on desulphurization by Hydrodynamic cavitation; (**a**) Pressure drop; (**b**) Initial concentration; (**c**) Volume fraction.

For the developed process, the effect of parameters such as pressure drop, initial concentration of the sulphur compound, organic volume fraction and nature of organic phase/solvent is believed to be most crucial and some important results pertaining to these are given in Fig. 4. Further details on the results of the above parameters are given in supplementary materials (Supplementary Figs S1–S7). Pressure drop across the vortex diode or for that matter any cavitating device, is an important parameter that contributes towards the extent of cavitation. The number density of cavities and effective intensity of cavity collapse are governed by pressure drop across cavitating devices (for a specified configuration of device and downstream piping). It is evident from Fig. 4, that the effect of pressure drop was found to be rather negligible, especially at low values of organic to aqueous phase volume ratio. Influence of initial concentration on sulphur removal is also shown in Fig. 4. It was observed that sulphur removal was generally better when initial sulphur concentration was low for high organic phase volume. The trend depends on nature of organic solvent and ratio (Fig. 4), though as seen from the figures, a very high removal close to 100% can be obtained at low organic fraction of 2.5%. In the developed process, there are two distinct liquid phases viz. aqueous and organic (containing sulphur compounds). Influence of nature of organic phase is expected to be important. Realized cavities and cavity collapse intensities are expected to vary with respect to the nature of organic phase and volume ratio of organic to aqueous phase. Experiments were therefore carried out for different organic solvents and using different volume ratios of organic to aqueous phase. Sample of results obtained with three different organic phases for different organic volume fractions are shown in Fig. 4. It can be seen that the removal of sulphur compounds is drastically different with different organic phases. A very high removal of sulphur was observed with n-octanol compared to other solvents. The order of sulphur removal with respect to the three organics studied in this work was as below:


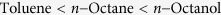


The initial concentration of sulphur in the organics and effectiveness of the developed process at low pressure drop have important bearing from the view point of commercial operations. Since conventional hydrodesulphurization process has limitations in bringing down sulphur levels below 350 ppm, a process that can take care of sulphur removal from this point can be highly beneficial. The results of this work clearly indicate effectiveness for initial concentrations up to 500 ppm. Further, effective deep desulphurization at low pressure drop of just 0.5 bar across the cavitating device indicates lower cost of operation and significant ease of operation. Another important aspect of the process is that it is effective without using any catalyst or requiring high temperatures/pressures.

**Figure 5:**
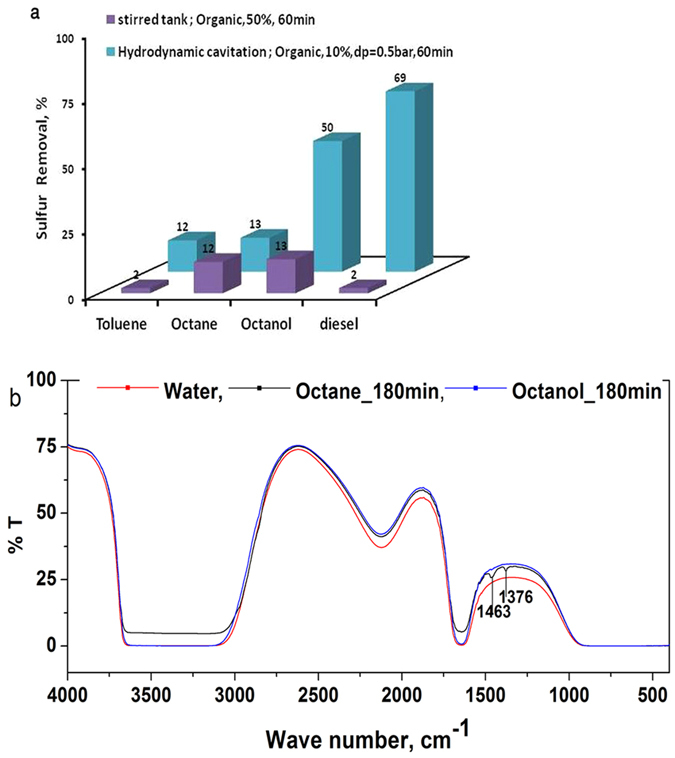
Cavitation is essential for reduction of sulphur content; (**a**) Sulphur removal using cavitation(Initial S: 300 ppm); (**b**) FTIR spectra of aqueous phase after cavitation.

It is also important to note that the insolubility of the thiophene in water and the huge difference with respect to the organic solvent practically eliminates possibility of physical transfer of the thiophene in the aqueous phase. In order to establish that sulphur removal is because of hydrodynamic cavitation and not because of vigorous contact with aqueous phase, the results of stirred tank experiments were also compared. The measured sulphur content of the different organics samples collected from these tanks (Initial sulphur concentration: 300 ppm;without cavitation) and those using hydrodynamic cavitation are shown in Fig. 5.

Hydrodynamic cavitation is known to generate hydroxyl radicals through cleaving of water molecules- an active oxidant and this can be effectively utilized for removal/degradation of organics^30^,^31^. A plausible mechanism for the removal of sulphur would require cleavage of the sulphur bond with the attack from the oxidant and release of sulphur dioxide. Alternatively, it can also form other oxidation products such as sulphone that would go in the aqueous phase, thereby effecting sulphur removal. A postulated mechanism can be thought to involve mainly reaction of the free radicals with the sulphur compounds resulting into removal of sulphur as SO_2_ and mineralization of the organic skeleton to final products as carbon dioxide and water.

It is also possible that the cavitation here works as a specific form of extractive, but not catalytic, oxidative desulphurization with water as a solvent and without employing any conventional catalyst of the type reported in the literature for ECOD. A number of other possibilities such as formation of SO_2_, HSO_3_, H_2_SO_4_ etc. may also be listed. No change in the pH of the aqueous solution was observed during or after hydrodynamic cavitation experiments, indicating that formation of acid or acidic species may not occur. The aqueous phase after the cavitation was analysed for increase in the organic carbon using COD/TOC test. It was observed that when toluene was used as the organic phase in cavitation, there was practically no increase in the COD/TOC values during the progress of cavitation indicating that no organic species entering into the aqueous phase due to cavitation. However, there was increase in the COD/TOC when n-octane was used as solvent in cavitation experiments, though the COD values were not high, but showed approximately 4 times increase (from 130 ppm to 650 ppm) indicating presence of organics in the aqueous phase which can be attributed to mainly organic solvent though possibility of entering solubilised forms of organic sulphur (e.g. sulphones) in the aqueous phase cannot be ruled out. Preliminary analysis of organic phase using gas chromatography, however, indicated no presence of any other compound than the solvent or thiophene. FTIR analysis of the aqueous samples (Fig. 5) indicated no appreciable presence of sulfones in the aqueous phase.

**Figure 6:**
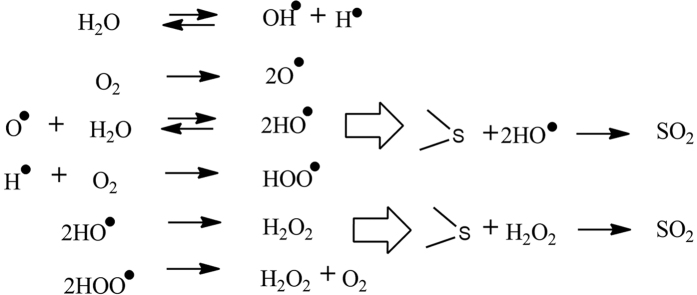
Proposed cavitative oxidation mechanism for desulphurization.

Details of mechanisms have been reported in the case of oxidative desulphurization in presence of various catalysts such as hydrogen peroxide and other acid catalysts^26^,^27^. The conventional approach involves reaction of hydrogen peroxide with the acid resulting into formation of acid peroxide which subsequently reacts with the organic sulphur resulting into formation of sulfone or sulfoxide. Otsuki *et al*.^27^ reported formation of sulfones in the organic phase during oxidative desulphurization using IR spectra analysis. The sulfone/sulfoxide, thus formed, can be extracted in different phase. It is important to note that the authors indicated difficulty in oxidising thiophene at 50 °C due to low electron density though benzothiophene or dibenzothiophene could be easily oxidised using hydrogen peroxide and formic acid mixture as catalyst. Thus, in the developed method, removal of organic sulphur is possible by both mineralization as well as oxidation mechanism (Fig. 6). However, since the process here does not employ acid catalyst, the contribution of later mechanism may not be significant. In principle, the cavities of organic, water or both (mixture) can form, the contribution of which would depend on the nature, vapour pressure of the organic solvent and composition. In the present work, the solvents, including water have vapour pressure in the range of 0.2–38 mm Hg at 30 °C; e.g. octanol (0.21), octane (17.65), toluene (37.7) and water (32) while thiophene has vapour pressure close to 92.6 mm Hg at 30 °C. However, in view of very low mol fraction of thiophene, its partial vapour pressure in the aqueous-organic two phase mixture of this study is significantly low. The probability of cavity formation for any phase is expected to increase with increase in its relative volume percent. However, even if organics cavities are more in number on the basis of low vapour pressure of the solvent, the degradation of sulphur species is believed to be difficult, if oxidative reaction by hydroxyl radical is assumed. The mechanism here is certainly much more complex and needs to be investigated in detail. In this context also, it is instructive to evaluate inception of the cavitation under different conditions/solvents and this can be done using the data of Fig. 3 for water alone and using different solvents for the two solvent ratios. The data on inception of cavities with addition of different solvents and with different ratios did not reveal significant difference from that of water alone, indicating that the role of solvent can be viewed predominantly as facilitator in oxidative interfacial reactions through effective transfer of sulphur moiety in cavities that provide predominantly oxidising species. However, this again needs further detailed investigation.

### Results with diesel

**Figure 7:**
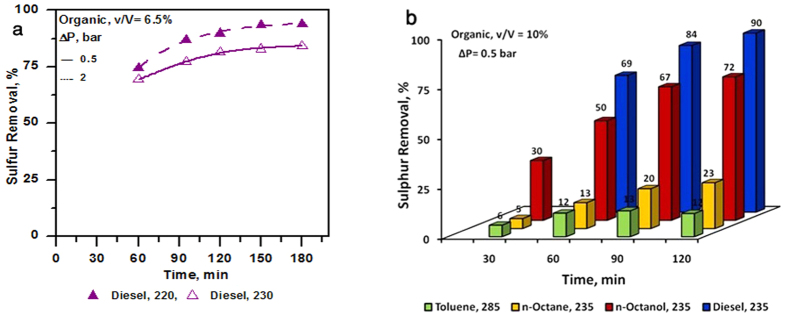
Comparison of deep desulphurization results using commercial diesel; (**a**) commercial diesel; (**b**) comparison with other organics.

It is instructive to evaluate the impact of nature of solvent from commercial application point of view for deep desulphurization of fuels. For this purpose, a commercial diesel was tested for the removal of sulphur (thiophene) similar to that discussed in the study. The commercial diesel had initial sulphur content of 30 ppm (probably in the form of refractory sulphur compounds). A known amount of sulphur using thiophene was added in this diesel and the effect of cavitation process was studied for pressure drop and for the extent of sulphur removal. The results of sulphur removal (thiophene in diesel) for the two different pressure drop conditions and using an intermediate organic to aqueous ratio (Organic phase, 6.5%) are shown in Fig. 7 along with a comparison with other organic solvents (Fig. 7). The results indicate a very high removal of sulphur even from the commercial diesel which is a mixture of aliphatic and aromatic organic compounds. Similar to that observed in certain cases earlier, here, the removal was better at higher pressure drop condition of 2 bar as compared to 0.5 bar. The results clearly demonstrate that proposed method is successful in removing sulphur from commercial diesel.

The proposed method can be effectively employed to reduce sulphur content of transportation fuels or other organic streams. The aqueous phase used in the proposed method can be recycled after removing a purge stream (with corresponding make-up water). The proposed method can be implemented in a compact set-up with effective removal of sulphur. The process may be further intensified using a number of ways e.g. aeration, catalyst etc. Hydrodynamic cavitation usually improves performance with scale-up. Therefore the proposed method can be effectively implemented for large scale deep desulphurization operation.

## Conclusions

A new multiphase non-catalytic process is developed for deep desulphurization of fuels or organics using hydrodynamic cavitation with vortex diode as a cavitating device. The process can completely remove thiophene sulphur from organic streams with considerable ease of operation and under mild operating conditions. Deep desulphurization of fuels to the extent of 100% was demonstrated for thiophene in model fuel. The removal efficiency depends strongly on nature of organics e.g. alcohols, aromatic solvents, aliphatic solvents or their mixtures apart from organic to aqueous ratio, pressure drop, and initial concentration of sulphur. A very high sulphur removal using commercial diesel was also demonstrated.

The aqueous phase used in the proposed method can be recycled after removing a purge stream (with corresponding make-up water). Hydrodynamic cavitation usually improves performance with scale-up and hence the proposed method can be effectively implemented for large scale deep desulphurization operations.

## Additional Information

**How to cite this article**: Suryawanshi, N. B. *et al*. A Non-catalytic Deep Desulphurization Process using Hydrodynamic Cavitation. *Sci. Rep.* **6**, 33021; doi: 10.1038/srep33021 (2016).

## Supplementary Material

Supplementary Information
