# Supplementary material for: A Non-catalytic Deep Desulphurization Process using Hydrodynamic Cavitation
Source: Sci Rep. 2016 Sep 8;6:33021. doi: 10.1038/srep33021 (PMC5015108; doi:10.1038/srep33021)
Supplement: Supplementary Information [file srep33021-s1.pdf]

Supplementary Information

A Non-catalytic Deep Desulfurization Process using Hydrodynamic Cavitation

Nalinee B. Suryawanshi, Vinay M. Bhandari*, Laxmi Gayatri Sorokhaibam and Vivek V. Ranade

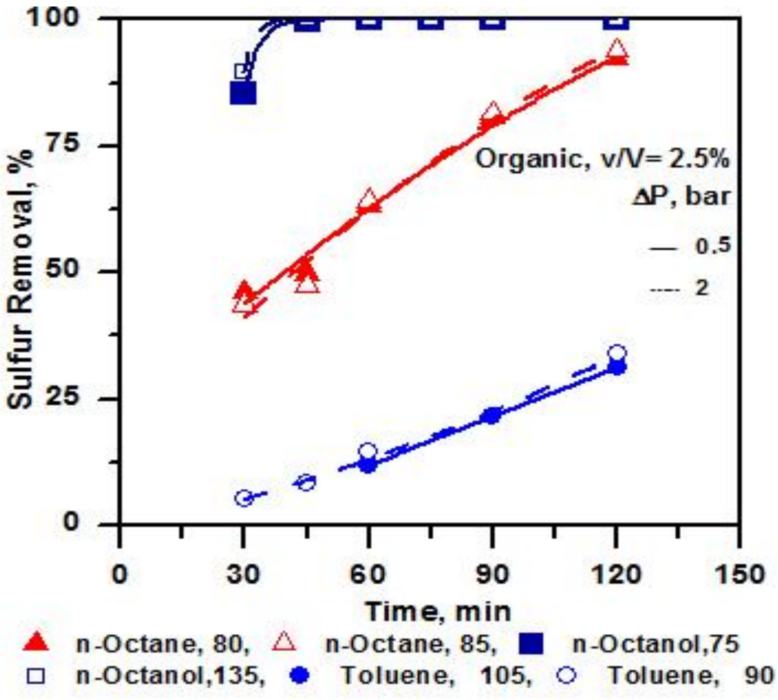

Supplementary Fig. S1. Effect of pressure drop at low initial sulphur concentrations

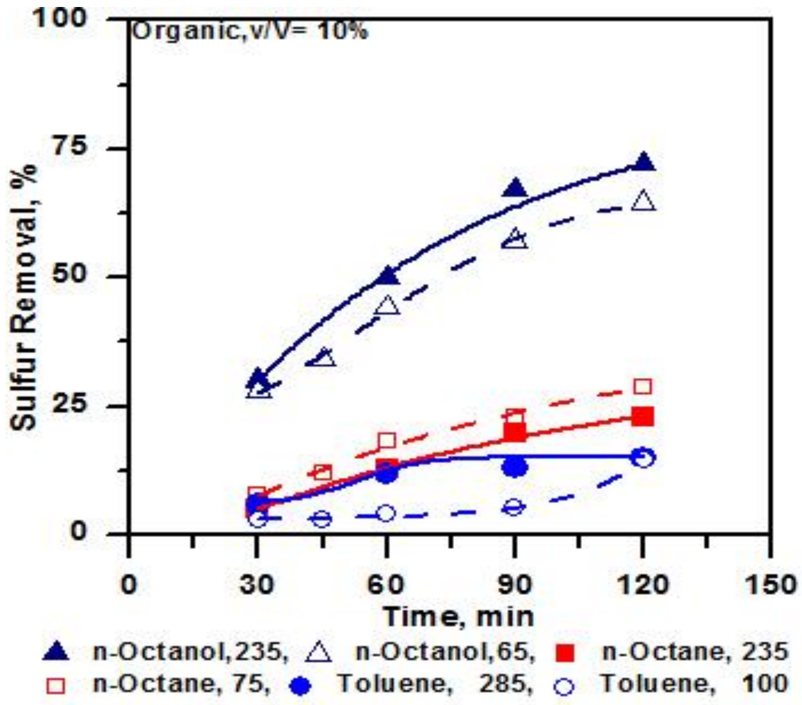

Supplementary Fig. S2. Effect of initial concentration at 10% organic phase volume (ΔP= 0.5 bar)

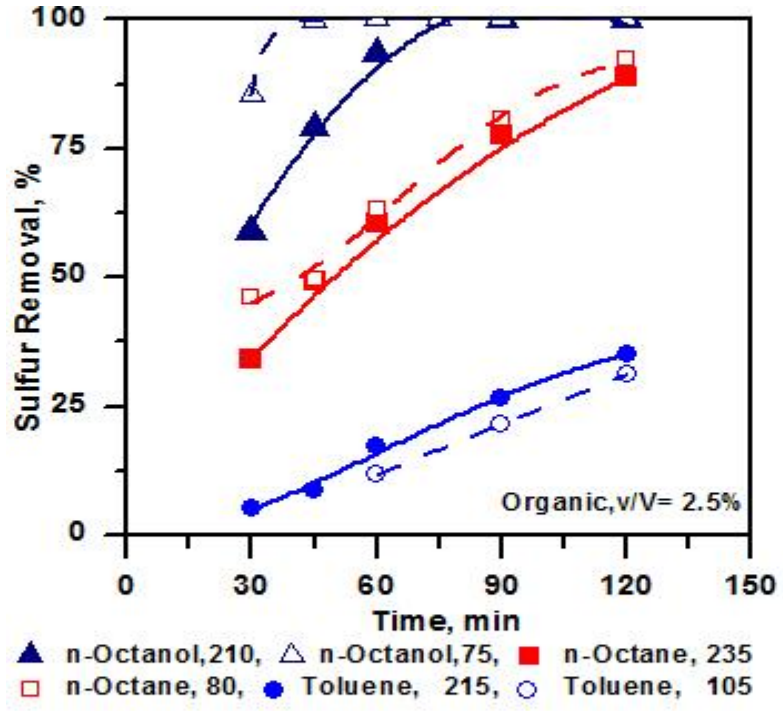

Supplementary Fig. S3. Effect of initial concentration at 2.5% organic phase volume (ΔP= 0.5 bar)

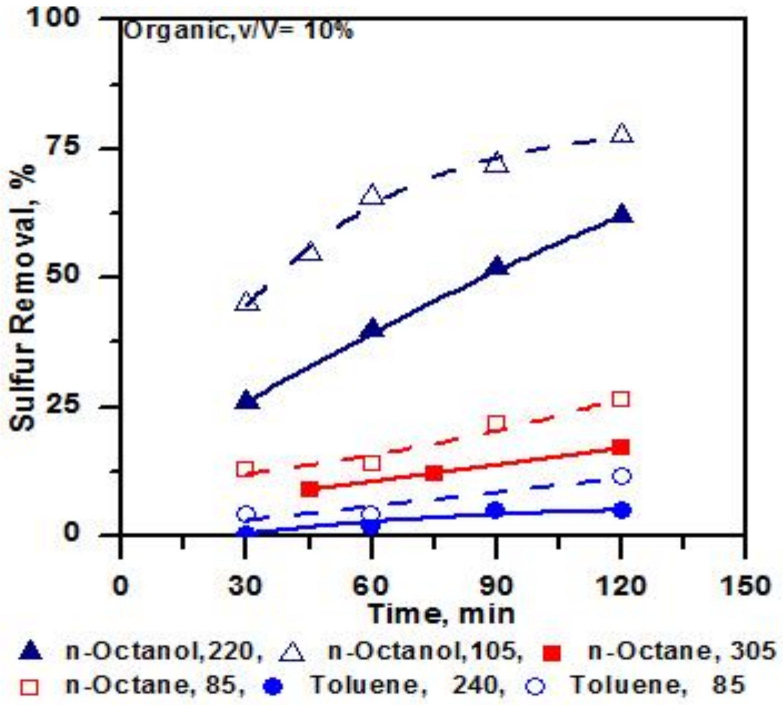

Supplementary Fig. S4. Effect of initial concentration at 10% organic volume (ΔP= 2 bar)

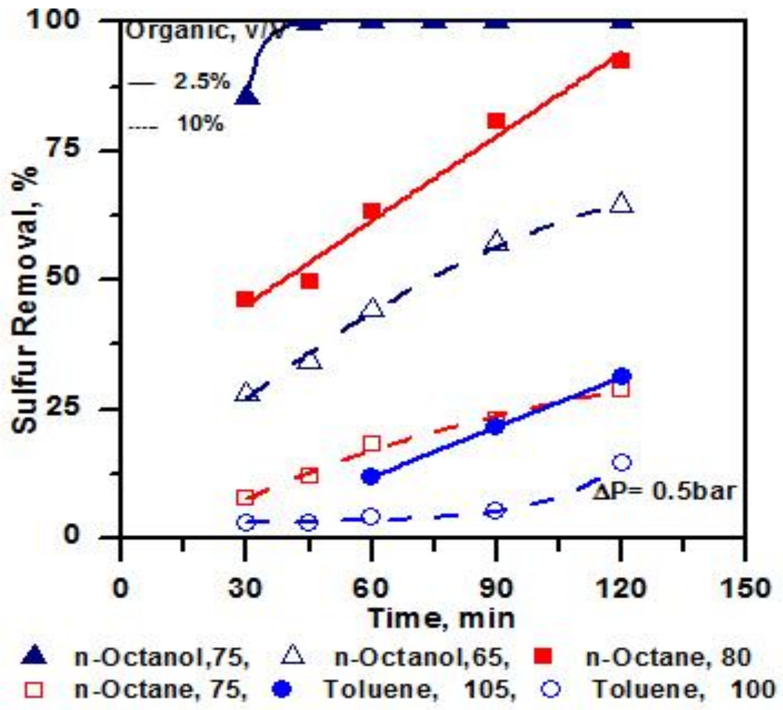

Supplementary Fig. S5. Effect of solvent ratio on sulphur removal at low initial concentrations (ΔP= 0.5 bar)

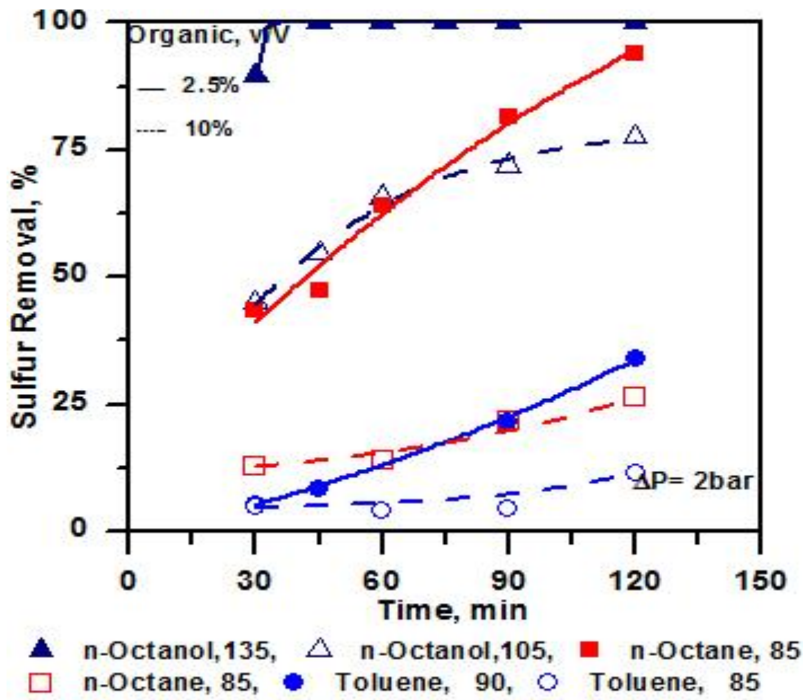

Supplementary Fig. S6. Effect of solvent ratio on sulphur removal at low initial concentrations (ΔP= 2 bar)

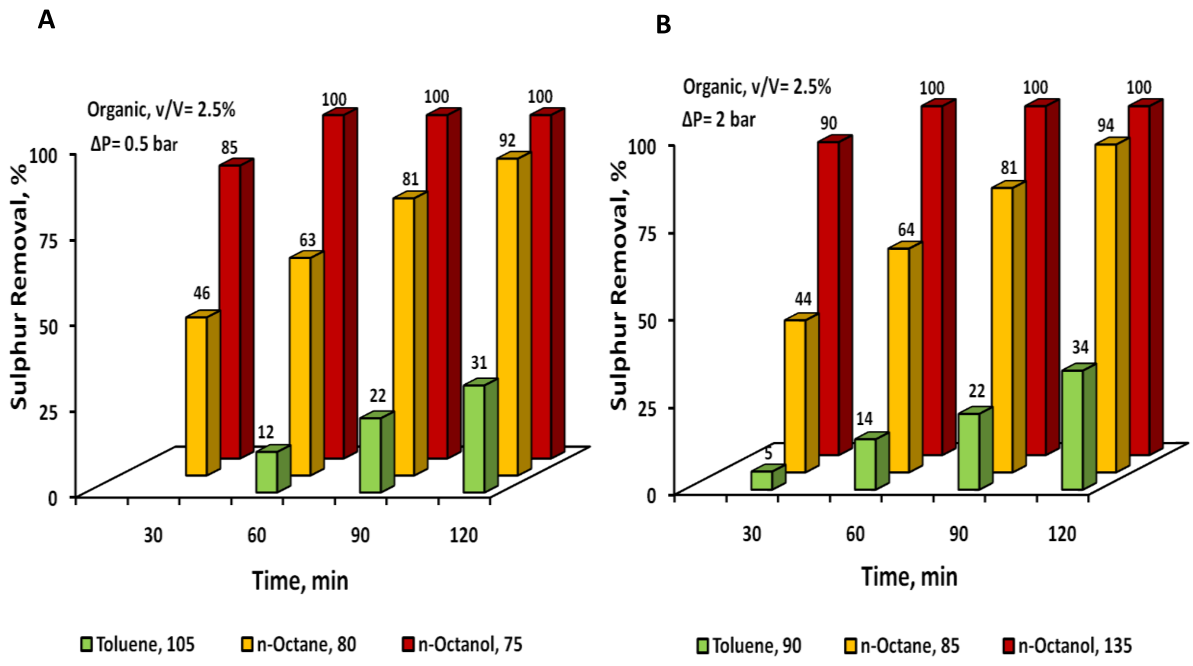

Supplementary Fig. S7. Comparison of effect of nature of solvent for low initial sulfur concentrations.

(A) ΔP= 0.5 bar. (B) ΔP=2 bar
